# Impaired DNA Replication within Progenitor Cell Pools Promotes Leukemogenesis

**DOI:** 10.1371/journal.pbio.0030401

**Published:** 2005-11-15

**Authors:** Ganna Bilousova, Andriy Marusyk, Christopher C Porter, Robert D Cardiff, James DeGregori

**Affiliations:** **1**Department of Biochemistry and Molecular Genetics, University of Colorado Health Sciences Center, Aurora, Colorado, United States of America,; **2**Program in Molecular Biology, University of Colorado Health Sciences Center, Aurora, Colorado, United States of America,; **3**Department of Pediatrics, University of Colorado Health Sciences Center, Aurora, Colorado, United States of America,; **4**Center for Comparative Medicine and Department of Pathology, School of Medicine, University of California, Davis, California, United States of America,; **5**Integrated Department of Immunology, University of Colorado Health Sciences Center, Aurora, Colorado, United States of America; Fred Hutchinson Cancer Research CenterUnited States of America

## Abstract

Impaired cell cycle progression can be paradoxically associated with increased rates of malignancies. Using retroviral transduction of bone marrow progenitors followed by transplantation into mice, we demonstrate that inhibition of hematopoietic progenitor cell proliferation impairs competition, promoting the expansion of progenitors that acquire oncogenic mutations which restore cell cycle progression. Conditions that impair DNA replication dramatically enhance the proliferative advantage provided by the expression of Bcr-Abl or mutant p53, which provide no apparent competitive advantage under conditions of healthy replication. Furthermore, for the *Bcr-Abl* oncogene the competitive advantage in contexts of impaired DNA replication dramatically increases leukemogenesis. Impaired replication within hematopoietic progenitor cell pools can select for oncogenic events and thereby promote leukemia, demonstrating the importance of replicative competence in the prevention of tumorigenesis. The demonstration that replication-impaired, poorly competitive progenitor cell pools can promote tumorigenesis provides a new rationale for links between tumorigenesis and common human conditions of impaired DNA replication such as dietary folate deficiency, chemotherapeutics targeting dNTP synthesis, and polymorphisms in genes important for DNA metabolism.

## Abstract 2

The authors show that the replicative competence of normal body cells can influence the rate of outgrowth of cells that carry an oncogene, helping explain a poorly understood aspect of carcinogenesis.

## Introduction

Despite significant advances in leukemia research, the factors that promote the selection and expansion of mutated cells leading to leukemia are not well known. Leukemias represent abnormal and poorly regulated hematopoiesis, with leukemia stem cells capable of self-renewal as well as the generation of multiple cell types [1]. Acquired mutations contributing to leukemogenesis, including chromosomal translocations that generate oncogenic fusion genes, may need to occur in hematopoietic stem cells (HSCs), as these cells have sufficient lifespan necessary for the acquisition of additional mutagenic hits. Alternatively, it is possible that oncogenic events happen in more committed progenitors, particularly if the mutation confers self-renewal on the cell or if the cell (such as a B cell progenitor) exhibits a sufficient life span [1,2].

Chronic myelogenous leukemia (CML) is a clonal myeloproliferative disease characterized by excessive proliferation of progenitor cells with massive accumulation of mature myeloid lineage cells. Untreated, the chronic phase is typically followed by an accelerated phase and blast crisis [3]. The t(9:22) Philadelphia chromosome translocation product generating p210 Bcr-Abl is the major cause of CML, and translocations generating either p210 or p190 *Bcr-Abl* fusions are present in childhood and adult acute lymphoblastic leukemias (ALLs). The CML-associated p210 *Bcr-Abl* gene is found in myeloid, erythroid, and lymphoid lineage cells in CML patients, suggesting that the translocation originally occurred in a pluripotent stem cell [3]. Recent evidence suggests that while the p210 *Bcr-Abl* fusion is found in HSC, the leukemia stem cells for both p210- and p190-associated ALLs possess committed B progenitor phenotypes [4]. The Bcr-Abl kinase promotes proliferation and growth factor independence, through the activation of a number of growth factor signaling pathways [5]. In addition, Bcr-Abl expression increases genomic instability, in part by stimulating homologous recombination repair, which can promote survival after DNA damage, while increasing recombination and loss of heterozygosity events [6].

A number of contexts associated with decreased cell cycle progression are, somewhat paradoxically, associated with high rates of tumors. The loss of the E2F1 and E2F2 transcription factors are generally associated with decreased proliferation in vitro and in vivo [7,8], consistent with their roles in promoting the transcription of a variety of genes required for cell cycle progression. Surprisingly, mice with mutations in *E2f1* and/or *E2f2* genes exhibit significantly increased cancer incidence [9,10]. Until now, the proposed tumor suppressive function of E2F1 and E2F2 has been attributed to decreased E2F-dependent apoptosis, DNA repair, or gene repression (reviewed in [11,12]). In addition, dietary folate deficiency, resulting in impaired DNA replication in cells in vivo, and polymorphisms that reduce dNTP synthesis are associated with increased colon cancer and leukemia rates [13,14]. Previous studies have substantiated that dNTP imbalances caused by folate deficiency or drugs that inhibit dNTP synthesis can increase DNA damage [14–16], and enhanced mutation accumulation is thought to underlie increased tumorigenesis [13,14]. Finally, patients treated with particular chemotherapeutic regimens, such as etoposide with methotrexate, are at increased risk of treatment-related acute myeloid leukemia [17]. Etoposide and methotrexate, which inhibit topoisomerase II and dihydrofolate reductase respectively, are effective inhibitors of DNA replication. Chemotherapeutics such as etoposide promote chromosomal translocations [17], and dNTP imbalances engendered by methotrexate may further promote mutation accumulation. Relevant to the studies presented here, genetic, dietary, and chemotherapeutic inhibitions of the dNTP and folate metabolic pathways are expected to promote replicative stress and DNA damage, leading to checkpoint activation and cell cycle inhibition.

While it is logical that conditions that negatively impact on a cell population could select for mutations conferring resistance, whether contexts of impaired proliferation contribute to tumorigenesis has not been previously demonstrated. We show that impaired DNA replication of hematopoietic progenitors in *E2f1/E2f2* mutant mice or mice treated with hydroxyurea (HU) allows Bcr-Abl-expressing and *p53*-mutated progenitors to outcompete nonmutated cells in the same niche, promoting leukemogenesis.

## Results

### The Poorly Replicating *E2f1* ^−/−^ *E2f2* ^−/−^ Progenitor Environment Favors the Competitive Expansion of Bcr-Abl-Expressing Progenitors

**Figure 1:**
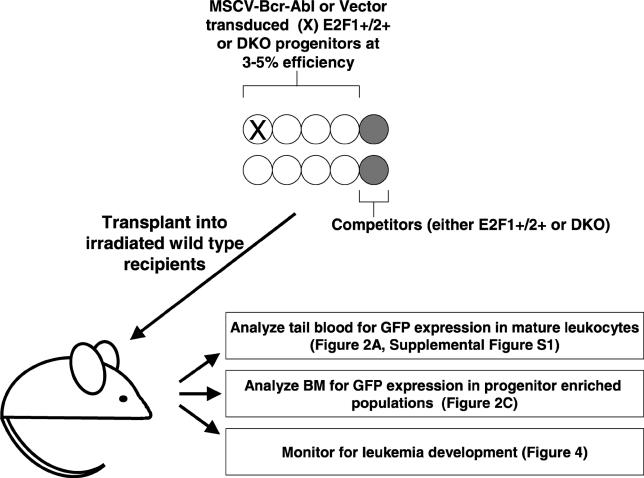
Experimental Design

DNA replication in bone marrow (BM) progenitor cell populations is severely impaired in *E2f1* ^−/−^ *E2f2* ^−/−^ (DKO) mice, but not in *E2f1* ^+/−^ *E2f2* ^+/−^ mice (phenotypically wild type; henceforth called *E2f1* ^+^ *2* ^+^), leading to severe hematopoietic deficiencies [7]. We tested the effects of *E2f1* and *E2f2* mutation on the expansion and leukemogenicity of cells expressing the p190 *Bcr-Abl* fusion oncogene, which is preferentially associated with ALL in humans [5]. For most experiments, we limited transduction with mouse stem cell viruses (MSCVs) encoding Bcr-Abl and/or green fluorescent protein (GFP) to only a small percentage of progenitors to more accurately reflect the rare occurrence of oncogenic initiating events in a background of unmutated cells, allowing for competition between *Bcr-Abl*-positive and -negative progenitor cells. The transduced cells were then transplanted into wild-type irradiated recipient mice, and competitive reconstitution was analyzed (see Figure 1 for the experimental design).

**Figure 2:**
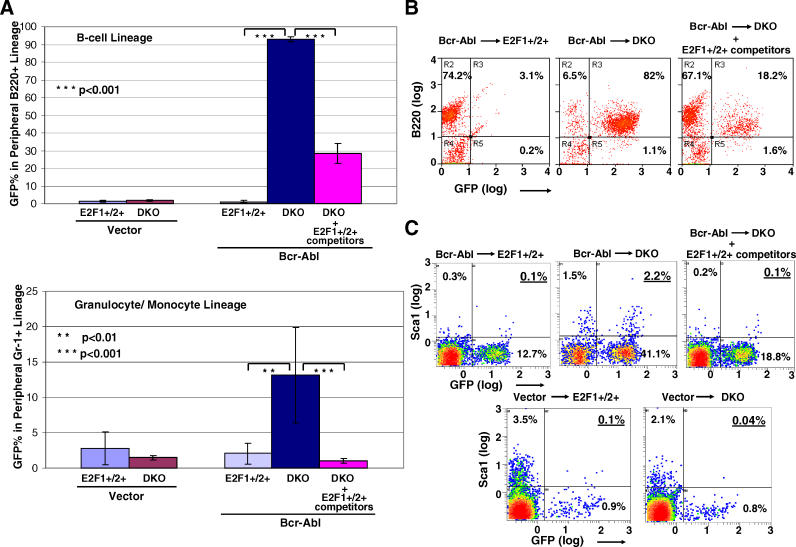
The Expression of Bcr-Abl Provides a Competitive Advantage to DKO Progenitors Purified c-Kit^+^ cells from either *E2f1* ^+^ *2* ^+^ or DKO donor mice (pooled sets of littermates) were transduced with MSCV-expressing p190 Bcr-Abl and GFP or GFP only (vector), and transplanted into lethally irradiated BALB/c recipients. The same pool of *Bcr-Abl* transduced DKO progenitors was either mixed with untransduced *E2f1* ^+^ *2* ^+^ competitor progenitors (“DKO + E2F1^+^2^+^ competitors”) or with untransduced DKO progenitors (“DKO”). *Bcr-Abl*-transduced *E2f1* ^+^ *2* ^+^ progenitors similarly were mixed with untransduced *E2f1* ^+^ *2* ^+^ progenitors (“E2F1^+^2^+^”). Specifically, each recipient received 2.5 × 10^5^ untransduced whole BM cells combined with 1.2 × 10^6^ MSCV-transduced c-Kit^+^ cells. Initial infection efficiencies measured for the whole c-Kit-enriched population were 5.1% and 3.75% among *E2f1* ^+^ *2* ^+^, and 4.73% and 3.4% among DKO progenitors for Bcr-Abl and vector, respectively. Tail blood isolated at 3 wk post-BMT was analyzed for the expression of GFP and B220 (upper bar graph; B-cell lineage) or GR-1 (lower bar graph; myeloid lineage) by flow cytometry. (A) Each bar represents data from five mice with standard error (SE) indicated, and statistical significance determined using Student's unpaired t-test (for all figures in this paper). (B) Representative flow cytometric profiles of peripheral B cells are shown, with the percentages of peripheral blood cells expressing both GFP and B220 indicated. (C) BM cells were analyzed by flow cytometry for the expression of Lin antigens, Sca1, Flk2 and GFP. Lin antigens included GR-1, B220, Ter119, CD3, and CD4. Cells analyzed in the profiles shown were gated as Lin^−^ Flk2^−^. The upper right quadrant represents progenitor-enriched Lin^−^ Flk2^−^ Sca1^+^ cells expressing either Bcr-Abl or vector, and the percentages of Lin^−^ Flk2^−^ cells that express Sca1 and/or GFP are indicated. In multiple experiments, recipients of *Bcr-Abl* transduced *E2f1* ^+^ *2* ^+^ cells exhibited reduced percentages of GFP^−^ Lin^−^ Flk2^−^ Sca1^+^ cells (the upper left quadrant) relative to vector controls, a phenomenon that we do not currently understand.

At low transduction efficiencies (less than 5% of transplanted cells expressing Bcr-Abl), we found that Bcr-Abl expression clearly provides a substantial advantage for DKO, but not *E2F1* ^+^ *2* ^+^ progenitors, as reflected by contributions to peripheral blood cells at 3 wk post-BM transplantation (BMT) (Figure 2A and 2B). The recipients of *Bcr-Abl-*transduced DKO cells had dramatically increased percentages of B cells and myeloid cells expressing Bcr-Abl. Increased contributions of Bcr-Abl-expressing cells in the DKO relative to *E2F1* ^+^ *2* ^+^ background were also observed at high infection efficiency, although a modest (but for many recipients, transient) advantage of Bcr-Abl expression was evident among *E2F1* ^+^ *2* ^+^ cells in terms of contributions to mature blood cells (Figure S1). As a control, we transduced BM progenitors with vector expressing GFP only, and in numerous experiments we found that *E2f1*/*E2f2* loss did not noticeably affect the ability to transduce stem and progenitor populations, as similar short-term and long-term reconstitution of all lineages as determined by GFP expression was observed in recipients of vector-transduced *E2f1* ^+^ *2* ^+^ and DKO BM progenitors (Figures 2A, 2C, and unpublished data).

In order to directly determine contributions to hematopoietic progenitor populations, we analyzed BM from the same set of mice for the expression of GFP (vector or Bcr-Abl) in particular progenitor pools. The lineage negative (Lin^−^) population in the BM expresses low levels of lineage-specific markers, and is thus enriched for progenitor cells. The Lin^−^ Flk2^−^ Sca1^+^ population is enriched for HSC but will also include short-term progenitors [18]. Of note, while Sca1 is expressed on only about 25% of HSC in BALB/c mice, the Lin^−^ Sca1^+^ population is substantially enriched for long-term HSC activity in these mice [19]. We observed a dramatic selective advantage for Bcr-Abl expression in the Lin^−^ Flk2^−^ Sca1^+^ population in the DKO, but not the *E2f1* ^+^ *2* ^+^, background (Figure 2C, upper right quadrants), correlating with contributions to peripheral blood cells. Of note, a transient advantage within more committed progenitors (such as Lin^−^ Sca1^−^ cells; Figure 2C, lower right quadrants) is often evident for Bcr-Abl expression in *E2f1* ^+^ *2* ^+^ cells. As we find that c-Kit is down-regulated on Lin^−^ Sca1^+^ cells post-BMT (unpublished data), we have been unable to further examine Bcr-Abl expression among highly defined HSC. In summary, Bcr-Abl expression provides a dramatic competitive advantage to progenitor cells in the replication-impaired DKO background, but not in the replication-competent *E2f1* ^+^ *2* ^+^ background.

### The Expression of Bcr-Abl Restores S Phase Progression in DKO Progenitor Cells

**Figure 3:**
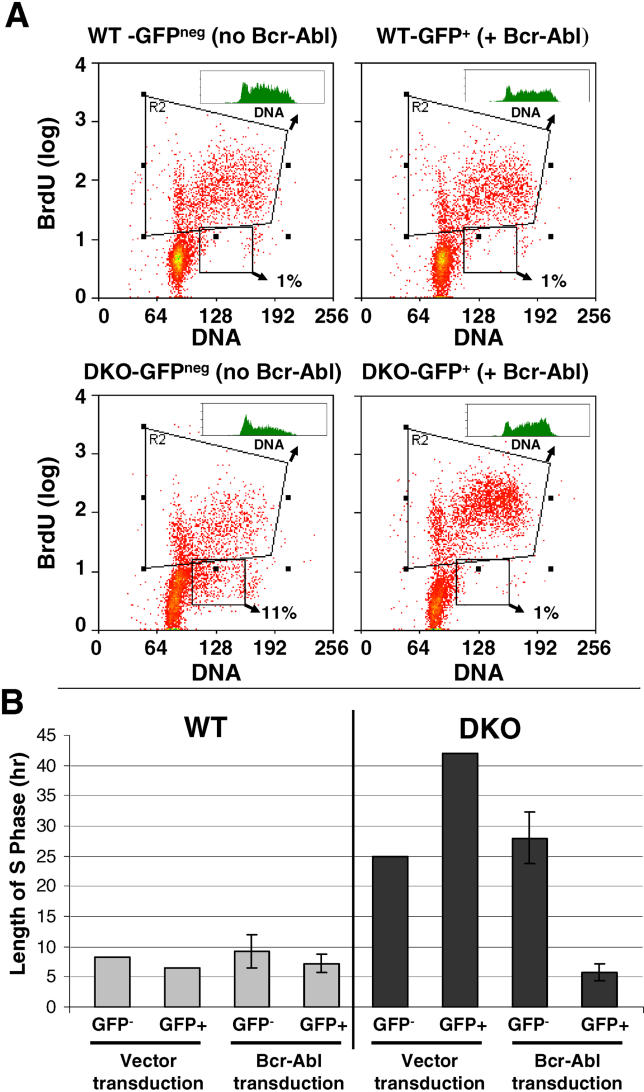
Bcr-Abl Restores S Phase Progression in DKO Progenitors Wild-type or DKO donor mouse c-Kit^+^ cells were transduced with MSCV-*Bcr-Abl* or vector and transplanted as in Figure 2 but at higher efficiency. (A) Recipients were injected 3 wk post-transplant with BrdU and spleen B cell and myeloid progenitors (combined) isolated by flow sorting, separating GFP^+^ from GFP^−^. Immunofluorescence for BrdU together with PI staining for DNA content are shown for GFP^−^ cells (internal control) and GFP^+^ cells from *E2f1* ^+^ *2* ^+^ or DKO recipients of *Bcr-Abl*-transduced stem cells. The percentages of cells with S phase DNA content but without significant BrdU incorporation (in the small square) are indicated. (B) The histograms shown in the insets in (A) plot the DNA content of cells gated as BrdU^+^, which are used to calculate the average lengths of S phase (from two experiments, ± SE).

The competitive expansion of Bcr-Abl-expressing cells relative to untransduced cells in the DKO background could be the result of overcoming cell cycle defects observed in DKO progenitors. To test this possibility, we assayed the effect of Bcr-Abl expression on S-phase progression rates in DKO progenitor cells using a bromodeoxyuridine (BrdU) pulse-chase method [20]. Two weeks post-BMT, at which point no signs of leukemia development were evident, mice were injected with BrdU and then sacrificed after 2 h. BrdU injection essentially provides a short pulse of the label, since BrdU half-life in vivo is very short [21]. Following flow cytometric isolation of GFP^+^ Lin^−^ or GFP^−^ Lin^−^ progenitors from the spleen, the average DNA content of BrdU-positive cells was assayed by propidium iodide (PI) intensity profiling. The rates of S phase progression were determined as the shift of the average PI intensity from the middle of S phase toward the G2 peak using formulas described by Begg et al. [20]. Although the calculated progression rates do not provide accurate absolute values for S phase length, since the presumption that the average PI intensity lies in the middle of S phase is not true for DKO cells (which accumulate in early S phase [7]), this method can still accurately analyze the changes of the rates caused by Bcr-Abl expression. Significantly, while S phase progression is substantially slowed in DKO progenitor cells, as previously reported [7], the expression of Bcr-Abl in these cells essentially restored S phase progression to control progression rates (Figure 3). In contrast, the expression of Bcr-Abl had no noticeable effects on S phase progression in wild-type progenitors. In addition, we previously showed that a fraction of DKO BM progenitors appear arrested in S phase, exhibiting S phase DNA content but no detectable BrdU incorporation [7]. As shown in Figure 3A, Bcr-Abl expression in DKO progenitors prevents the accumulation of this S phase-arrested population.

Thus, the competitive advantage provided by Bcr-Abl expression to DKO progenitors appears to derive from restored cell cycle progression. Furthermore, as we find that the expression of Bcl2 does not provide an advantage to DKO progenitors relative to wild-type progenitors (Figure S2), the ability of Bcr-Abl to block apoptosis does not appear to underlie the selective advantage conferred by Bcr-Abl expression in the DKO background, although decreased apoptosis could be a contributing factor.

### The DKO Background Promotes Bcr-Abl-Mediated Leukemogenesis

**Figure 4:**
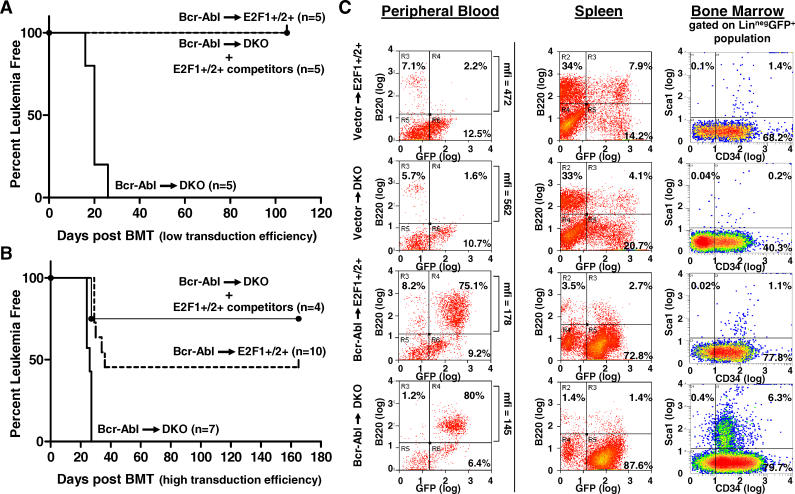
E2f1/*E2f*2 Loss Promotes Bcr-Abl-Mediated Leukemias (A and B) *E2f1* ^+^ *2* ^+^ or DKO mouse c-Kit^+^ cells were transduced with MSCV-*Bcr-Abl* and transplanted as in Figure 2, except that in (B) a higher initial transduction efficiency was obtained (23.2% for *E2f1* ^+^ *2* ^+^ and 24.1% for DKO). Initial transduction efficiencies for (A) were the same as in Figure 2. Untransduced competitors were included as described in Figure 2, except that for the experiments shown in (B), competitors were c-Kit^+^-purified BM cells cultured in parallel to the transduced cultures but not infected, and each recipient mouse received 1.5 × 10^5^ untransduced c-Kit^+^ competitors combined with 1.4 × 10^6^ *Bcr-Abl*-transduced progenitors. Kaplan-Meier curves are shown for both experiments. Mice were sacrificed when moribund, all with splenomegaly and lymphadenopathy. No mice transplanted with vector-expressing cells developed leukemia (unpublished data). The Kaplan-Meier curves for *Bcr-Abl*/DKO are statistically different from the *Bcr-Abl*/ *E2f1* ^+^ *2* ^+^ (*p* < 0.001 for [A] and *p* < 0.0001 for [B]) and *Bcr-Abl*/DKO + *E2f1* ^+^ *2* ^+^ competitors (*p* < 0.001 for [A], *p* < 0.02 for [B]) curves. (C) Peripheral blood, spleen, and BM from morbid leukemic *Bcr-Abl* BMT mice or from healthy vector BMT mice (from [B]) were analyzed for the expression of the indicated antigens and GFP by flow cytometry. Peripheral blood was analyzed 21 d post-BMT. The recipient of *Bcr-Abl*/DKO cells was sacrificed at 23 d and other recipients sacrificed at 29 d post-BMT. The mean fluorescence intensities (mfi) for peripheral B220^+^ cells are indicated.

As leukemogenesis appears to require initiating mutations in stem or progenitor cell populations, we addressed whether the greater competitive advantage provided to Bcr-Abl-expressing DKO progenitors translates to increased tumor incidence in transplanted mice. Strikingly, all recipients of *Bcr-Abl*-transduced DKO progenitors developed rapid pre-B ALL-like disease using both low and high initial transduction efficiencies (Figures 4A and 4B), preceded by rapid increases of peripheral GFP^+^ B220^+^ cells (Figures 2A and S1). In contrast, only about half of the recipients of *E2f1* ^+^ *2* ^+^ cells transduced at high efficiency, and none of the recipients at low transduction efficiency, developed leukemia (also pre-B ALL; Figure 4). For leukemias arising in both genetic backgrounds, leukemic infiltrates were evident in spleen, liver, and lung, and peripheral blood GFP^+^ cells expressed low B220, exhibiting a large, immature morphology with pale-staining nuclei (Figure 4C and unpublished data). Leukemia cells in the spleen and BM were mostly negative for the Lin markers GR-1, Ter119, CD4, and CD3 (unpublished data), expressing from undetectable to low B220 levels depending on the leukemias (Figure 4C; examples shown express little to no B220). Interestingly, we consistently observed a greater fraction of Sca1^+^CD34^+^ cells in DKO relative to *E2f1* ^+^ *2* ^+^ leukemias (Figure 4C), consistent with the early advantage provided by Bcr-Abl expression in DKO progenitor-enriched populations (Figure 2C).

### *E2f1* ^+^ *2* ^+^ Competitors Effectively Suppress Leukemia Development by Bcr-Abl-Expressing DKO Progenitors

While leukemogenesis correlates with the ability of Bcr-Abl-expressing cells to competitively expand within progenitor pools, based on increased leukemia rates alone we cannot distinguish whether or not cell autonomous effects of *E2f1/E2f2* loss, such as accelerated mutagenesis or decreased apoptosis, are the major factors underlying increased leukemia rates. To directly address this issue, we included *E2f1* ^+^ *2* ^+^ untransduced competitor cells with *Bcr-Abl*-transduced DKO progenitors; amazingly, these competitors were able to almost completely eliminate leukemogenesis (Figures 4A and 4B). *E2f1* ^+^ *2* ^+^ competitors also substantially reversed the selective advantage conferred by Bcr-Abl expression in the DKO background as reflected in peripheral blood cells (Figures 2A, 2B, and S1) and within stem cell-enriched populations (Figure 2C). Similarly, transplantation of *Bcr-Abl*-transduced DKO progenitors into sublethally irradiated recipients resulted in the rebound of host (wild-type) hematopoiesis and complete prevention of leukemias (unpublished data). Thus, enhanced leukemogenesis is not primarily due to cell autonomous effects of *E2f1* and *E2f2* loss, but most likely results from the poor ability of DKO progenitor cells to compete with Bcr-Abl-expressing DKO progenitors.

Importantly, these results suggest that the failure of many recipients of *Bcr-Abl*-transduced *E2f1* ^+^ *2* ^+^ (particularly at low transduction efficiency) to develop leukemia was not due to our inability to introduce *Bcr-Abl* into the appropriate progenitor cells, but to the inability of *Bcr-Abl* ^+^ progenitors to be maintained in the face of replication competent competitors. Finally, while it remains formally possible that DKO stem/progenitor cells home poorly to BM niches and that Bcr-Abl restores homing to DKO progenitors, the fact that a low percentage of cotransplanted *E2f1* ^+^ *2* ^+^ BM can reverse the selective expansion of *Bcr-Abl* ^+^ DKO progenitors and leukemogenesis (Figures 4 and S1) strongly argues against a role for differential homing in genotype-dependent leukemia rates.

In summary, a DKO progenitor pool promotes Bcr-Abl-dependent leukemogenesis due to the inability of untransduced DKO progenitors to provide effective competition. The ability of Bcr-Abl to restore S phase progression to replication impaired DKO progenitors most likely underlies the competitive advantage provided by Bcr-Abl specifically within the DKO background.

### HU Promotes the Competitive Expansion of Bcr-Abl-Expressing Blood Progenitors Ex Vivo

**Figure 5:**
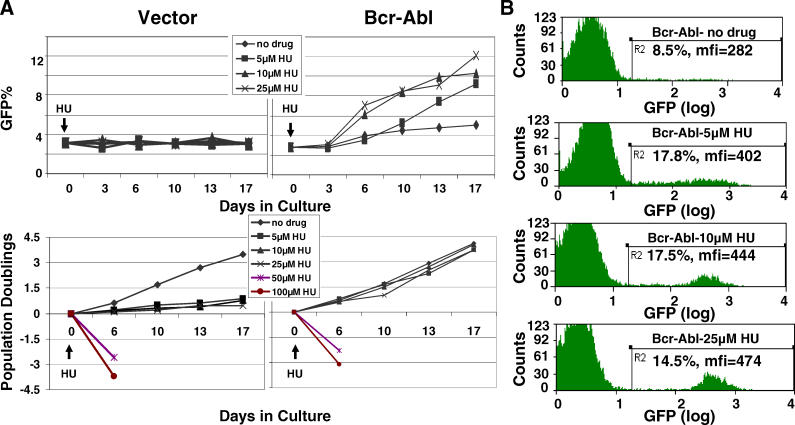
HU Promotes the Competitive Expansion of *Bcr-Abl* ^+^ Blood Progenitors Ex Vivo (A) BALB/c c-Kit^+^ BM cells were transduced with MSCV-*Bcr-Abl* or vector and cultured in stem cell medium with HU (0, 5, 10, 25, 50, or 100 μM) added starting at day 3. Flow cytometric analysis of GFP expression and total cell counting in cultures were performed every 3–4 d. The changes in GFP percentages in vector and Bcr-Abl cultures (upper graphs) and the cumulative expansion (log_e_ scale) of *Bcr-Abl* ^+^ and vector cells (lower graphs) during HU treatment were plotted. (B) The percentages of cells expressing Bcr-Abl (day 13) as well as the mean fluorescence intensities (mfi) for the GFP^+^ population, which has been shown to reflect Bcr-Abl protein expression [35], are indicated. Most *Bcr-Abl* ^+^ cells were c-Kit^+^CD34^+^, with or without HU treatment. HU treatment did not affect the intensity of GFP in vector cells (unpublished data).

In order to further explore the generality of the selection for Bcr-Abl-expressing cells under conditions of impaired DNA replication as well as to examine Bcr-Abl-mediated leukemogenesis in a more clinically relevant context, we asked how the suppression of proliferation within hematopoietic progenitor pools during treatment of mice with HU, an inhibitor of ribonucleotide reductase, affects Bcr-Abl-dependent competition within wild-type BM. HU has been used for over 40 y as a cytostatic drug [22]. HU-induced dNTP depletion results in replication stalling and the activation of checkpoint pathways [23]. We initially asked whether HU treatment creates a poorly competitive environment that favors the expansion of Bcr-Abl-expressing blood progenitors ex vivo. As shown in Figure 5A, HU differentially affected Bcr-Abl- and vector-expressing progenitors, allowing *Bcr-Abl* ^+^ cells to competitively expand. In fact, while the expansion of vector-expressing progenitors was almost completely inhibited by all concentrations of HU used, Bcr-Abl-expressing progenitors continued expanding at doses ranging from 5 to 25 μM (Figure 5A, lower panels). Higher doses of 50 and 100 μM HU did effectively inhibit the expansion of *Bcr-Abl* ^+^ progenitors, indicating that the advantage provided by Bcr-Abl expression during HU treatment is relative but not absolute. While Bcr-Abl expression alone provided a modest advantage to wild-type progenitors in culture, 5 to 25 μM HU treatment conferred an even greater advantage to Bcr-Abl-expressing cells (Figure 5A, upper panel). Furthermore, as determined by GFP expression levels, HU selects for cells expressing higher levels of Bcr-Abl (Figure 5B). Notably, increased Bcr-Abl expression is commonly observed in patients during CML progression from chronic phase to blast crisis [24].

### HU Treatment Provides a Competitive Advantage to Bcr-Abl-Expressing Progenitors In Vivo

**Figure 6:**
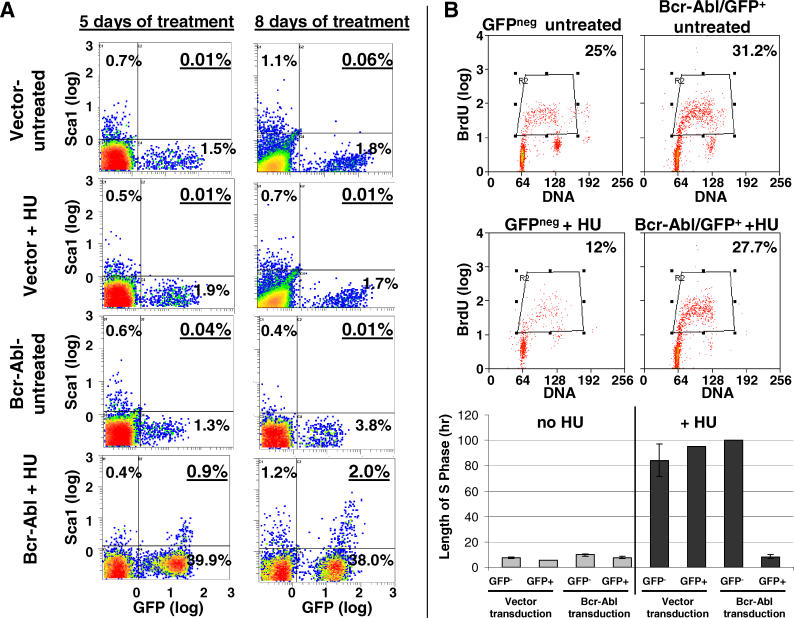
HU Selects for Bcr-Abl Expression Within Hematopoietic Progenitor Pools In Vivo BALB/c c-Kit^+^ BM cells were transduced with the indicated retroviruses, and transplanted into sublethally irradiated BALB/c recipients. At 2 wk post-BMT, half of each group was switched to water containing HU. (A) BM cells were analyzed for the expression of Lin antigens, Sca1, and GFP as described in Figure 2C on days 5 and 8 of HU treatment. Cells analyzed in the profiles shown were gated as Lin^−^. The percentages of the progenitor enriched Lin^−^ Sca1^+^ population that expressed vector or Bcr-Abl are indicated in the upper right corners (underlined). (B) Four days after switching to HU water (or not), mice were injected with BrdU, and spleen GFP^+^ and GFP^−^ Lin^−^ progenitors isolated by flow sorting as in Figure 3. BrdU incorporation is detected in GFP^−^ cells (internal control) and GFP^+^ cells, and the calculated average lengths of S phase from two experiments is shown in the graph.

Mice were transplanted with MSCV-p190 *Bcr-Abl*-transduced BALB/c BM progenitor cells, and half of the recipients were switched to water containing HU (20 mg/kg/d; similar to doses used clinically for cytoreduction [22]) at 2 wk post-BMT. Strikingly, by 5 and 8 d after switching recipient mice to HU, a substantial competitive advantage for *Bcr-Abl* ^+^ cells within the progenitor-enriched Lin^−^ Sca1^+^ population was evident, while a similar selection for Bcr-Abl expression was not observed without HU treatment (Figure 6A). Note that about two-thirds of the progenitor-enriched Lin^−^ Sca1^+^ cells expressed Bcr-Abl in HU-treated mice, while very few *Bcr-Abl* ^+^ Lin^−^ Sca1^+^ cells were detected in untreated mice, despite the fact that these mice were transplanted with the same pool of transduced BM progenitors. The rapid competitive advantage provided by Bcr-Abl specifically following HU treatment in vivo, together with the almost immediate selection for Bcr-Abl expression by HU ex vivo, argues that HU-induced mutagenesis does not account for the effects of HU on the competitive expansion of *Bcr-Abl* ^+^ progenitors.

To understand why Bcr-Abl provides such an advantage specifically following HU treatment, we analyzed cell cycle progression in Bcr-Abl-expressing progenitors in vivo using the same method used for Figure 3. While HU decreased the percentage of cells undergoing DNA synthesis and increased the length of S phase, Bcr-Abl expression resulted in more normal S phase progression in leukocytic progenitors in HU-treated recipients (Figure 6B). This result provides a mechanistic insight into why HU treatment improves the competitive expansion of Bcr-Abl-expressing progenitors in vivo and ex vivo: While HU impedes the proliferation of competing progenitors, Bcr-Abl overcomes this block. In contrast, Bcr-Abl expression does not appear to affect DNA replication in untreated progenitors, and thus Bcr-Abl expression does not provide an apparent competitive edge without HU.

### HU Treatment Accelerates and Increases the Penetrance of Bcr-Abl-Mediated Leukemias

**Figure 7:**
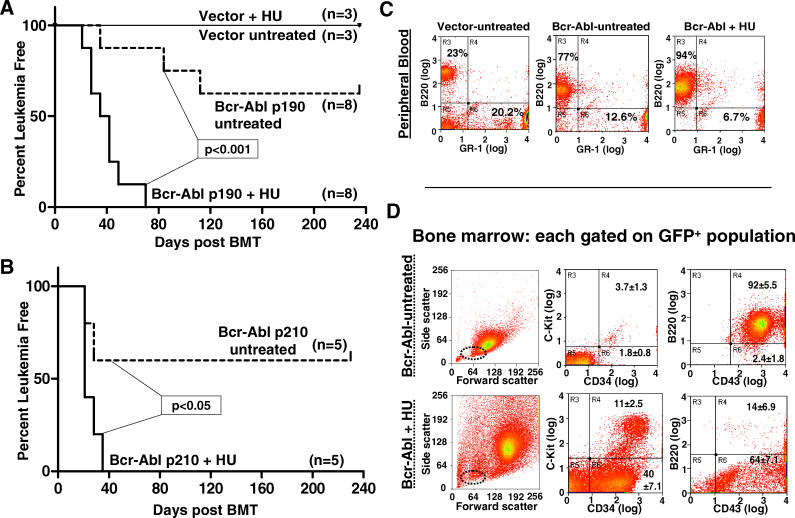
HU Treatment Enhances Bcr-Abl-Mediated Leukemogenesis *Bcr-Abl* BMT recipients were generated and treated with HU as described in Figure 6. (A–B) Kaplan-Meier curves for vector (A), p190 *Bcr-Abl* (A), or p210 *Bcr-Abl* (B) transduced stem cell-transplanted mice with or without HU treatment are shown. The curves for p190 and p210 *Bcr-Abl* with HU are statistically different from the respective *Bcr-Abl*-untreated curves (*p* < 0.001 and *p* < 0.05, respectively). For (A) and (B), the average percent GFP^+^ in peripheral blood cells was 3%, and mice with similar percentages were segregated into the HU-treated and untreated groups. Mice were sacrificed when moribund, all with splenomegaly and massive increases in B220^+^GFP^+^ (for p190) or GR-1^+^GFP^+^ (for p210) cells in the spleen and peripheral blood. (C) Peripheral blood from morbid p190 *Bcr-Abl*-BMT mice (with or without HU, as in Figure 6) or from a healthy vector BMT mouse was analyzed for B220 and GR-1 expression by flow cytometry. (D) BM cells from morbid p190 BMT mice were analyzed for the expression of c-Kit, CD34, CD43, B220, with side scatter and forward scatter (reflects cell size) as indicated. The dashed oval indicates the typical position of normal BM pre/pro-B-cells. All leukemic BM cells in untreated *Bcr-Abl* recipients expressed B220 and CD43 as shown in the upper panels. Leukemic BM cells from HU treated mice generally expressed less B220 (despite B220 expression in peripheral cells; see (A) and displayed more c-Kit^+^ and many more CD34^+^ cells. Percentages shown are averages (± SD) for three mice for each group. Two *Bcr-Abl* ^+^ mice treated with HU that developed leukemia late (7 and 10 wk post-BMT) displayed an immunophenotype more similar to the *Bcr-Abl*-untreated mice.

We addressed whether HU treatment of mice with *Bcr-Abl* ^+^ progenitors promotes leukemogenesis in vivo. For each experiment, aliquots from a single pool of MSCV-*Bcr-Abl* (or vector)-transduced BALB/c BM stem cells were transplanted into multiple syngeneic recipients. For all recipients, ∼3% of peripheral B cells were GFP^+^ at 2 wk post-BMT, at which point half of the mice were switched to water containing HU. By treating with HU starting 2 wk post-BMT, potential effects of HU on retroviral infection efficiency or homing to BM niches are avoided. Strikingly, all of the HU-treated mice developed a pre-B ALL-like disease within 2 mo (Figure 7A) preceded by increases in the percentages of *Bcr-Abl* ^+^ cells in the B220^+^ subset (unpublished data). In contrast, mock-treated mice that received *Bcr-Abl*-transduced stem cells developed leukemia with much lower penetrance (3 of 8) and longer latency periods. HU treatment also enhanced leukemogenesis induced by p210 Bcr-Abl, although interestingly, these mice all succumbed to CML-like disease (Figure 7B). Thus, in our model system, p190 Bcr-Abl causes B-ALL and p210 causes CML, thus closely modeling the human diseases caused by the respective translocation products. Given that HU treatment promotes the expansion of *Bcr-Abl* ^+^ progenitors within 5 d (Figure 6A), it is improbable that HU-promoted mutagenesis alone accounts for more rapid disease, although increased mutation accumulation may be a contributing factor. Furthermore, as HU similarly promotes leukemogenesis in analogous experiments using donor BM from *Rag2* ^−/−^ mice, in which case mature T and B cells were nearly undetectable in recipients (Figure S3), HU-mediated increases in Bcr-Abl-dependent leukemogenesis does not appear to result from immunosuppression.

Leukemic p190 *Bcr-Abl* recipient mice exhibited massive increases in pre-B-like large B220^+^ CD43^+^ cells, high leukocyte blast counts in peripheral blood, and splenomegaly (Figures 7C and S4). Pathological characterization of spleens and other tissues determined that the lymphoproliferative diseases arising in all HU and the three non-HU-treated mice were large cell leukemias, with frequent involvement of liver, lung, and spleen (unpublished data). Spleens contained a mixture of leukemic cells, with most exhibiting lymphoid characteristics and a subset exhibiting a myeloid phenotype (unpublished data). For most HU (relative to non-HU)-treated mice, the predominant GFP^+^ (i.e., *Bcr-Abl* ^+^) population in the BM and spleen contained a more substantial subpopulation of cells expressing the progenitor markers c-Kit and CD34 (Figure 7D). We transplanted spleen or BM cells (10^3^–10^5^) from morbid leukemic HU- and non-HU-treated *Bcr-Abl* BMT recipients into BALB/c recipients, and most recipients for both groups developed leukemia, underscoring the malignant nature of the leukemias (Figure S5). Thus, by providing a competitive advantage to *Bcr-Abl* ^+^ progenitor cells (as shown ex vivo and in vivo), HU treatment promotes Bcr-Abl-mediated ALL and CML.

### The Inhibition of p53 Provides a Competitive Advantage to DKO BM Progenitors

**Figure 8:**
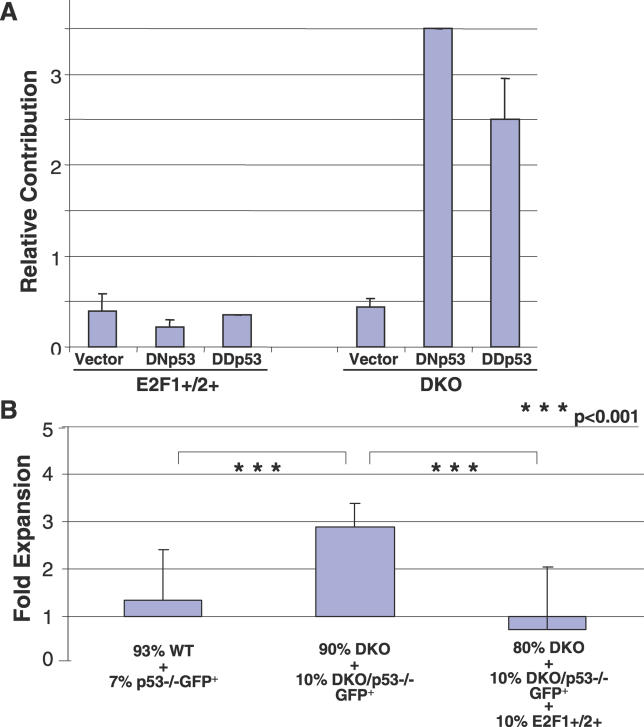
The Inhibition of p53 Provides a Competitive Advantage for DKO, but Not Wild-Type, BM Progenitors (A) Wild-type or DKO BM cells were transduced with MSCV-DNp53, MSCV-DDp53, or vector, and transplanted into lethally irradiated BALB/c recipients. At 2 wk post-BMT, the percentage of GFP^+^ cells among peripheral blood GR-1^+^ cells was determined by flow cytometry. The contribution of GFP^+^ cells after 2 wk relative to the initial infection efficiency is shown. Initial infection efficiencies for each virus were similar for the two BM genotypes. (B) Genetic disruption of *p53* provides a proliferative advantage in the DKO but not wild-type background. The indicated mixtures of freshly harvested BM were used to reconstitute the hematopoietic system of lethally irradiated recipient BALB/c mice. At 6 wk post-BMT, the percentage of GFP^+^ nucleated cells in peripheral blood was determined by flow cytometry and fold expansion was determined relative to the initial mixture.

While our experiments provide strong evidence that backgrounds of impaired DNA replication enhance the competitive expansion of Bcr-Abl-expressing cells, we wanted to test if this phenomenon can be applicable to other oncogenic events. The *p53* tumor suppressor gene is mutated in over half of human tumors, and mutation of *p53* has been shown to disrupt checkpoints that prevent cell cycle progression in the face of various cellular stresses [25]. Mutation of *p53* in human cancers frequently results in the expression of a dominant negative mutant p53 protein (DNp53) that forms inactive multimers with wild-type p53. Given the known ability of DNp53 to alleviate checkpoint-dependent cell cycle arrests, we asked whether the expression of DNp53 (175H) could provide a competitive advantage for *E2f1/E2f2* mutant progenitor expansion. In addition, we engineered a retrovirus to express the oligomerization domain of p53 (DDp53), which also inhibits p53 activity [26]. Strikingly, MSCV-mediated expression of DNp53 or DDp53 in hematopoietic progenitors from DKO, but not wild-type, mice resulted in a dramatic selective advantage for these cells after only 2 wk post-BMT (Figure 8A). Again, the percentages of peripheral blood cells expressing vector were very similar for wild-type and DKO cells. Thus, the inhibition of p53 provides a competitive advantage during hematopoiesis in DKO, but not wild-type, BM.

We used genetic disruption of *p53* [27] as an alternative to retroviral DNp53 expression. We bred the *p53* mutation into the *E2f1/E2f2* mutant and BALB/c backgrounds together with a ubiquitously expressed GFP transgene [28]. In competitive BMT experiments using wild-type BM in competition with *p53* ^−/−^GFP^+^ BM (93:7 ratio), *p53* ^−/−^GFP^+^ BM contributions to hematopoiesis were similar to its percentage within the original BM transplanted (Figure 8B), indicating that *p53* mutation does not provide an immediate competitive advantage during hematopoiesis. In contrast, DKO/*p53* ^−/−^GFP^+^ BM progenitors exhibited significant competitive expansion relative to DKO progenitors, and this competitive expansion was completely abrogated by the inclusion of *E2f1* ^+^ *2* ^+^ competitors (Figure 8B; compare second and third columns). Thus, as in the Bcr-Abl experiments shown in Figure 2, the increased expansion of DKO/*p53* ^−/−^ cells in a DKO background relative to *p53* ^−/−^ cells in a wild-type background is not due to cell autonomous contributions of *E2f1/E2f2* loss, but instead to the poor competition provided by DKO hematopoiesis.

## Discussion

Given that deregulated cell cycling is considered a hallmark of tumorigenesis [29], it is natural to assume that contexts that promote proliferation would promote cancer. Studies in search of connections between genetic polymorphisms, dietary factors, and environmental exposures that predispose to cancer are often predicated on the assumption that increased cancer rates in part result from the enhancement of proliferation by these conditions [30–33]. We show that the opposite can be true. Our data indicate that the reduced ability of progenitor cell pools to proliferate actually promotes tumorigenesis, in that cells acquiring mutations that improve proliferation outcompete poorly replicating competitors. In contrast, a replication-competent pool is inherently tumor-suppressive, and otherwise oncogenic mutations may not be favored.

We have shown that within a wild-type, healthy progenitor cell pool, Bcr-Abl expression or p53 inhibition does not provide an apparent selective advantage. In contrast, in contexts of impaired DNA replication of blood progenitors, expression of Bcr-Abl or inhibition of p53 provides an immediate advantage within progenitor pools. The expression of Bcr-Abl bypasses the S phase blocks imposed by *E2f* mutation or HU treatment, consistent with the known ability of Bcr-Abl to abrogate cell cycle checkpoints. As Bcr-Abl expression provides an immediate competitive advantage following HU treatment ex vivo, the advantage appears to result from the better proliferation of *Bcr-Abl* ^+^ cells relative to untransduced peers, not effects of HU on mutagenesis or the immune system, or other potential complications found in vivo. Most importantly, replication-impaired contexts (both genetic and chemotherapeutic) can substantially promote Bcr-Abl-dependent leukemias. That Bcr-Abl-expressing cells with restored S phase progression outcompete untransduced cells with highly impaired replication provides a logical explanation for the promotion of leukemogenesis by HU treatment and *E2f1/E2f2* loss. Indeed, the promotion of Bcr-Abl-dependent leukemias by *E2F* mutation is not due to cell autonomous contributions of *E2f1/E2f2* loss, as *E2f1* ^+^ *2* ^+^ competitors virtually eliminate leukemias resulting from Bcr-Abl expression in DKO progenitors. Thus, healthy competitors are potently tumor-suppressive.

**Figure 9:**
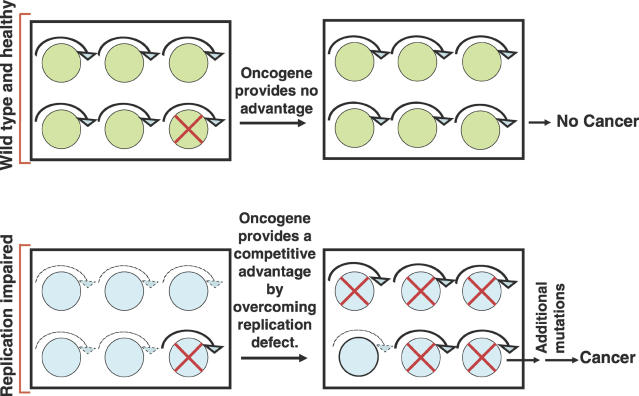
The Poor Competition Model In a wild-type, healthy progenitor cell pool, potentially oncogenic mutations (represented as a red “X” in a cell) will often provide little or no advantage in the short term, and may even be disadvantageous (top images). In contrast, in a replication-impaired hematopoietic system (bottom images), acquisition of some oncogenic mutations should provide a selective advantage by partly or fully restoring cell cycle progression, even when the same mutation might be disadvantageous in a wild-type pool. The preferential expansion of the mutated population increases the target size and thus the chances for additional mutations and cancer. In this figure, the rectangle indicates the limited niche size, implying competition for contacts and growth factors. The thickness of curved arrows indicates proliferative ability.

The presented results support a model whereby the reduced proliferation of blood progenitors provides a powerful selection for oncogenic mutations that improve cell cycle progression. In essence, impeded proliferation of progenitor cells provides for poor competition, promoting the expansion of progenitors that acquire oncogenic mutations which relieve cell cycle blocks (Figure 9). In addition, the ability of some oncogenic mutations to impede apoptosis resulting from checkpoint activation might further contribute to a net competitive advantage. It has been previously proposed that carcinogen-mediated inhibition of cell survival or proliferation can select for oncogenically mutated cells resistant to these conditions ([34] and references therein). Analogously, we show that conditions that impair DNA replication in the entire population of progenitor cells select for oncogenic mutations that initiate tumorigenesis. In contrast, oncogenic mutations prove less advantageous or even disadvantageous in a normal hematopoietic system, as cell cycle progression in the progenitor pool is already efficient, and checkpoints should not be activated. Finally, contexts that involve nucleotide deprivation and DNA damage, and thus impeded DNA replication, may enhance mutation accumulation, and mutator phenotypes should synergize with poor competition to promote tumorigenesis. Thus, these conditions provide not only an increased chance for the acquisition of initiating mutations, but also a poorly competitive environment that favors the expansion of these oncogenically mutated cells. In this regard, it is unlikely that all conditions that globally impair cell cycle progression in a stem cell pool will promote tumorigenesis, particularly if these impaired contexts cannot be easily overcome by oncogenic mutation or do not sufficiently promote mutation accumulation. Moreover, while impaired replication can clearly select for the expression of oncogenes such as Bcr-Abl and DNp53, other oncogenic events (like Bcl2 overexpression) may not be selected for and may even be selected against.

The low leukemia incidence that we observed in the replication competent-backgrounds may seem at odds with previous reports of efficient induction of CML-like disease in recipients of MSCV-*Bcr-Abl*-transduced BM cells using high-efficiency infections [35–37]. So why would MSCV-*Bcr-Abl* transduction result in rapid and penetrant onset of leukemia with high- but not low-efficiency infection, given that normal hematopoietic stem and progenitor cells, and thus healthy competitors, are still present in both cases? One possibility is that the resulting high percentage of cells expressing Bcr-Abl could result in substantial autocrine stimulation, which may not reflect the normal context of a rare *Bcr-Abl* fusion event. Indeed, expression of Bcr-Abl in cytokine-dependent cell lines leads to growth factor-independent proliferation associated with autocrine production of interleukin-3 (IL-3) and granulocyte/monocyte-colony stimulating factor [38–40], and increased levels of IL-3 are detected in serum in murine models of CML [36,37]. Importantly, retroviral expression of IL-3 by BM cells is sufficient to induce a myeloproliferative syndrome in mice [41–43]. Furthermore, *Bcr-Abl* ^+^ cells secrete inhibitors of normal hematopoiesis that induce apoptosis in normal but not *Bcr-Abl* ^+^ cells [44]. Autocrine stimulation by Bcr-Abl-expressing cells and inhibition of normal hematopoiesis should have a far greater impact when a large percentage of cells express Bcr-Abl, as compared to the physiological context of an initiating mutation leading to the expression of the oncogene in a single clone. Nonetheless, these activities could still be very relevant to the progression of *Bcr-Abl* ^+^ leukemias, in that once a *Bcr-Abl* ^+^ clone has reached some critical mass, the clone may promote its own competitive expansion even in the face of normal hematopoietic competitors. Finally, high-efficiency transduction of progenitors should increase the chance of secondary mutations that promote Bcr-Abl-dependent leukemogenesis, including gene alterations mediated by retroviral integrations.

An important point is that most of the recipients of control (*E2f1* ^+^ *2* ^+^ or wild-type without HU) progenitors transduced with Bcr-Abl did not develop leukemia. Indeed, for surviving mice, GFP expression in peripheral blood became undetectable (unpublished data), indicating that Bcr-Abl expression is disfavored in stem cell pools in the long-term. The facts that (1) the same pool of *Bcr-Abl*-transduced BM progenitors was transferred into recipients that subsequently were mock- or HU-treated, and (2) all HU-treated *Bcr-Abl* recipients rapidly developed leukemia, indicate that most of the nonleukemic mice in the control group did receive *Bcr-Abl* ^+^ progenitors capable of causing leukemia, but that these cells were not maintained. In contrast, HU treatment clearly promoted the expansion of *Bcr-Abl* ^+^ cells, stimulating leukemogenesis. Analogously, *Bcr-Abl*, *IgH-Bcl2*, and *LMO2-TCR* translocations are frequently found in nonleukemic individuals who do not subsequently develop leukemias [45]. Based in part on the detection of Bcr-Abl in peripheral blood of healthy people for several months to years, an argument has been made that *Bcr-Abl* translocations must occur frequently in HSCs of people that never develop leukemia [46]. In light of the data presented here, we suggest that the relative inability of progenitor cells possessing *Bcr-Abl* translocations to competitively expand and to acquire additional mutations under most contexts limits the development of leukemia.

Our result that HU promotes Bcr-Abl-dependent leukemias may seem surprising, given that HU is used for cytoreduction of CML cells in patients. However, in human disease the leukemic cells are already established and dominant, while our experiments are more relevant to the context of leukemia initiation. Thus, our use of HU may be less pertinent to the use of this drug to treat CML, but can be viewed as a general means to suppress S phase progression in the hematopoietic system, which is relevant to the effects of clinically used drugs or other conditions that inhibit DNA replication in progenitor populations. Indeed, a number of contexts that have been presumed or demonstrated to reduce dNTP production are associated with increased cancer rates, including folate deficiency [14] and polymorphisms resulting in decreased thymidylate synthase expression [47]. These contexts of dNTP restriction are thought to promote cancer in part by increasing mutation rates, and our results suggest that poorly competitive stem cell pools also play an important role. While links between *Bcr-Abl* ^+^ CML or ALL incidence and dietary variables or polymorphisms affecting dNTP metabolism have not been sufficiently examined, based on our demonstration that the inhibition of dNTP synthesis by HU promotes Bcr-Abl-dependent leukemias, such studies are warranted.

HU induces replication-associated double-stranded DNA breaks in cultured cells [15,16], although whether HU induces DNA damage or mutagenesis in vivo is still unresolved. Long-term treatment of sickle cell anemia patients with HU does not result in increased mutation rates or cancer formation [48]. However, some studies have found an association between cytoreductive HU treatment of myeloproliferative disorders and the development of acute leukemias, including a high proportion with 17p deletion [49,50]. Still, while commonly used chemotherapeutic antimetabolites such as HU and methotrexate do not appear to be substantially leukemogenic when used as single agents [48,51], combinations of antimetabolites with etoposide are associated with higher incidences of treatment-related leukemias relative to etoposide alone [17]. While clinical experiences are insufficiently controlled to conclude that antimetabolite therapies contribute to secondary leukemias, our demonstration that a chemotherapeutic agent that inhibits dNTP synthesis can promote leukemia suggests that this issue needs to be further addressed in the clinic. The inhibition of DNA replication by antimetabolites may promote the competitive expansion of progenitor cells acquiring etoposide-induced oncogenic mutations, much as HU promotes the expansion of Bcr-Abl-expressing progenitors. Thus, conditions that generally impair DNA replication, such as drugs that inhibit dNTP synthesis, may function as tumor promoters for oncogenically initiated cells by creating a poorly competitive environment. This view contrasts with the generally described function of tumor promoters as enhancers of proliferation.

Finally, while numerous properties of E2F1 and E2F2, including gene repression and the promotion of apoptosis and DNA repair, have been suggested to underlie observed increases in tumors in *E2f1* and *E2f2* mutant mice (reviewed in [11,12]), we demonstrate that the promotion of Bcr-Abl-dependent leukemias and the expansion of *p53* mutant cells by *E2f1/E2f2* disruption result from non-cell autonomous consequences of *E2f* loss. According to our model, *E2f1/E2f2* mutations at least in part predispose mice to tumors, since the *E2f* genes are disrupted in all cells in the mouse, thus providing a poorly competitive progenitor population. In contrast, loss-of-function mutations in *E2f1* or *E2f2* in an individual cell would not be expected to provide an advantage to the cell, and thus would not contribute to tumorigenesis, thus perhaps explaining the paucity of such mutations in human tumors. Poor competition should select for oncogenic mutations only when the entire population of competing cells are similarly debilitated.

In summary, we have demonstrated that impaired DNA replication in hematopoietic progenitor pools contributes to a powerful selection for mutations (such as Bcr-Abl translocation) that improve cell cycle progression, providing a selective advantage over the poor competition offered by replication-debilitated progenitors and thus promoting leukemogenesis. As cancer development has attributes clearly reminiscent of Darwinian evolution, it is logical that it should become easier for a mutant cell clone to be the “fittest” as the quality of the competition declines. Thus, as with the natural selection of species, poor fitness selects for adaptive mutations in stem cell pools.

## Materials and Methods

### Mice

The generation of the *E2f1* and *E2f2* mutant mice (backcrossed seven times into BALB/c) has been previously described [52,53]. BALB/c and *p53* ^−/−^ mice were purchased from Jackson Labs (Bar Harbor, Maine, United States). GFP transgenic mice were the generous gift of Dr. Brian Schaefer, and were backcrossed with *p53* mutation into the BALB/c background. The University of Colorado Health Sciences Center Animal Care and Use Committee approved all mouse experiments.

### Retroviral constructs

The retroviral MSCV-iresGFP vectors expressing either p190 or p210 Bcr-Abl were provided by Drs. Zhonghan Dai and Warren Pear. MSCV-*Bcl2*-iresGFP was provided by Dr. Scott Lowe. To construct similar retroviral vectors expressing DNp53 and DDp53, the p53Arg175His cDNA fragment from plasmid pCMV-NEO pC53–175 (provided by Dr. Bert Vogelstein) and DDp53 cDNA from pLXSNp53DD (provided by Dr. Xiao-Fan Wang) were cloned into the MSCV-iresGFP vector.

### Retroviral transduction and bone marrow transplantation

MSCV viruses were prepared by transient transfection of Phoenix-E cells together with pCL-Eco, and titered on fibroblasts using GFP as the marker, so that similar multiplicities of infection for oncogene and vector viruses were used. Donor DKO, *E2f1* ^+^ *2* ^+^, or wild-type mice were injected intraperitoneally with 100 mg/kg 5-fluorouracil (Sigma, St. Louis, Missouri, United States) 3 d prior to BM harvest. Harvested donor BM cells were enriched for c-Kit^+^ cells by MACS (Miltenyi Biotec, Bad Gladbach, Germany) and then cultured overnight in IMDM plus 15% defined fetal bovine serum (HyClone, South Logan, Utah, United States) and 0.1% bovine serum antigen (Sigma), with the cytokines 100 ng/ml human stem cell factor (gift from Dr. Chris Hogan), 100 ng/ml human IL-11 (PeproTech, Rocky Hill, New Jersey, United States),1.5 ng/ml mouse IL-3 (PeproTech), and 100 ng/ml hIL-6 (PeproTech). Cells were transduced with MSCV-containing supernatants (∼1/5 volume) supplemented with 6 μg/ml Polybrene (Sigma) for 6 h. Cells were then transplanted intravenously into lethally (1,200 Rads for E2F experiments) or sublethally (600 Rads for HU experiments) irradiated BALB/c mice. A subset of the transduced cells was further cultured for 2 d, and the percentage of GFP^+^ cells was determined by flow cytometry in order to determine the initial infection efficiency. Ex vivo culturing of *Bcr-Abl*-transduced cells for Figure 5 was performed under the same culture conditions.

### Flow cytometry and cell cycle analysis

Single-cell suspensions were washed in PBS containing 5% FBS (FBS-PBS). About 10^6^ cells were stained in 20 μl of antibody solution (1:100 dilution of each antibody) for 30 min at room temperature. Cells were washed once with 1 ml of FBS-PBS and resuspended in 400 μl of PBS for flow cytometric analysis. The following PharMingen (San Diego, California, United States) antibodies against mouse were used: phycoerythrin (PE)-linked anti-B220, PE-anti-Ter119, PE-anti-GR-1, PE-anti-CD3, PE-anti-CD4, PE-anti-Flk2/Flt3, and PE-anti-CD43; and allophycocyanin (APC)-linked anti-B220, APC-anti-Thy1.2, and APC-anti-CD117; and biotin-linked anti-GR-1, biotin-anti-CD34, biotin-anti-IgM, and PE-cyanine (PE-Cy7)-linked anti-biotin. APC-anti-Sca1 antibodies were purchased from eBioscience (San Diego, California, United States). Fluorescence was detected with a Cytomics FC 500 (Beckman Coulter, Allendale, New Jersey, United States) cytometer. For BrdU incorporation analyses, mice were injected intraperitoneally with BrdU (1 mg per 25 g of body weight; Roche, Indianapolis, Indiana). After 2 h, pooled spleen and BM cells were purified using a MoFlo (Cytomation, Fort Collins, Colorado, United States) cell sorter for GFP^+^ or GFP^−^ subsets of progenitors: B220^+^ plus GR-1^+^ (B-cell and myeloid lineages) for Figure 3 or Lin^−^ for Figure 6. Immunofluorescence, flow cytometric analyses, and calculations of the length of S phase were performed as described previously [7,20].

### Pathology

Mice were monitored for disease development, as judged by increasing percentages of GFP^+^ cells with blast morphology in peripheral blood of transplanted animals, as well as symptoms, such as abnormal gait and labored breathing. Moribund animals were sacrificed and examined for tumors. Touch preps and tissue sections were obtained from spleen and liver.

## Supporting Information

Figure S1 The Loss of *E2f1* and *E2f2* Promotes the Competitive Repopulation of the B Cell Lineage by Bcr-Abl-Expressing Progenitors C-kit^+^ cells from either *E2f1* ^+^ *2* ^+^ or DKO donor mice were transduced at low- and high-efficiency and transplanted as in Figures 2 and 4 (same experiments). The graph shows the changes in the percentages of GFP^+^ cells in the peripheral B220^+^ lineage of *Bcr-Abl* transduced *E2f1* ^+^ *2* ^+^E2F1^+^ recipients (left), *Bcr-Abl* transduced DKO recipients (middle) and *Bcr-Abl* transduced DKO recipients supplemented with untransduced *E2f1* ^+^ *2* ^+^ competitor progenitors (right). Each curve follows the individual mouse used in experiments with low (upper) and high (lower) transduction efficiency. Curves end if the mouse develops leukemia and is sacrificed prior to the next tail blood analysis. Surviving mice from the *Bcr-Abl*→*E2f1* ^+^ *2* ^+^ and *Bcr-Abl*→DKO + *E2f1* ^+^ *2* ^+^ competitors groups were bled at 180 and 240 d post-BMT (for low- and high-transduction experiments, respectively), and no GFP^+^ cells were detected above background in any recipient (unpublished data).
(54 KB PPT).

Figure S2 \Inhibition of Apoptosis by Bcl2 Overexpression Does Not Provide a Competitive Advantage to DKO Relative to *E2f1* ^+^ *2* ^+^ Progenitors *E2f1* ^+^ *2* ^+^ or DKO BM cells were transduced with MSCV viruses expressing Bcl2 (with iresGFP), and then transplanted into lethally irradiated BALB/c mice. The mice were sacrificed at 3 wk post-transplant, and flow cytometric analysis of GFP and GR-1 expression in BM was performed. Initial infection efficiencies for both genotypes were similar. MSCV-expressed Bcl2 is functional, given that it conferred increased survival to BM progenitor cultures from *E2f1* ^+^ *2* ^+^ and DKO mice (unpublished data).
(54 KB PPT).

Figure S3 HU Treatment Increases p190 Bcr-Abl-Mediated Leukemogenesis in the *Rag2* ^−/−^ Background (A) C-Kit^+^ cells from *Rag2* ^−/−^ BM (BALB/c congenic) were transduced with MSCV-p190 *Bcr-Abl*, and transplanted into lethally irradiated BALB/c recipients as in Figure 2. At 2 wk post-BMT, half of each group was switched to water containing HU. The average percent GFP^+^ in peripheral blood cells was 10%, and mice with similar percentages were segregated into the HU treated and untreated groups. Kaplan-Meier curves for transplanted mice are shown. Mice were sacrificed when moribund; all with splenomegaly and massive increases in GFP^+^ cells in the spleen and peripheral blood.
(B) Representative flow cytometric plots are shown for recipients of *Bcr-Abl*-transduced *Rag2* ^−/−^ BM. Peripheral blood cells of the transplanted mice were analyzed for the expression of CD3 and IgM 2 wk post-BMT (before HU treatment). Cells expressing these markers (T and B cells) were almost undetectable in transplanted animals (left). Spleen cells from morbid *Bcr-Abl*/*Rag2* ^−/−^ (with and without HU) mice were analyzed for the expression of GFP, B220, Ter119, GR-1, and Thy1.2. Leukemias arising in untreated and HU-treated mice did not express B220 and Thy1.2 (unpublished data), but displayed many GR-1^+^ and Ter119^+^ cells (right; gated on GFP^+^ blast population). A similar blast population expressing both myeloid (GR-1^+^) and erythroid (Ter119^+^) markers was present in the one morbid non-HU treated recipient (unpublished data).
(114 KB PPT).

Figure S4 Recipients of *Bcr-Abl*-Transduced Progenitors That Developed Leukemias With or Without HU Treatment Exhibited Splenomegaly and High Numbers of Leukocytic Blasts in Peripheral Blood Recipients of vector or p190 *Bcr-Abl* transduced progenitors were treated or not with HU as in Figure 7 and sacrificed when morbid. Peripheral blood from the tail vein was drawn before sacrifice and analyzed for a complete blood count using a Cell-Dyn 1700 System. HU treatment of vector recipients did result in modest but significant (*p* < 0.05) leukopenia. For *Bcr-Abl* recipients with or without HU treatment, high peripheral leukocyte counts were evident, most of which were blasts (also confirmed microscopically), but the difference between HU-treated and untreated was not statistically significant. Following sacrifice, spleens were weighed. The increased spleen weight in HU-treated relative to untreated recipients of *Bcr-Abl* transduced progenitors is significant (*p* < 0.05). The average weight of a spleen from a healthy mouse is about 0.1 g.
(40 KB PPT).

Figure S5 Leukemias That Developed in Untreated and HU-Treated Animals Were Transferable BM cells (5 × 10^3^, 5 × 10^4^, and 5 × 10^5^) from morbid leukemic HU and non-HU treated *Bcr-Abl* BMT recipients were transplanted into BALB/c recipients. The fraction of transplanted mice that developed secondary leukemias is shown, and the time post-transplant that mice became morbid indicated in parentheses. Similar results were obtained with spleen cells from a different set of leukemic donors.
(36 KB PPT).

### Accession Numbers

The GenBank (http://www.ncbi.nlm.nih.gov/Genbank/) accession numbers of the genes discussed in this paper are *Bcr-Abl* (X06418), *E2f1* (L21973), *E2f2* (AK087452), and *p53* (P04637).

## Abbreviations

ALL: acute lymphoblastic leukemia
BM: bone marrow
BMT: bone marrow transplantation
BrdU: bromodeoxyuridine
CML: chronic myelogenous leukemia
DDp53: oligomerization domain of p53
DKO: E2f1^−/−^E2f2^−/−^
DNp53: dominant negative p53 mutant
E2f1^+^2^+^: E2f1^+/−^E2f2^+/−^
GFP: green fluorescent protein
HSC: hematopoietic stem cell
HU: hydroxyurea
IL: interleukin
Lin−: lineage negative
MSCV: mouse stem cell virus
PI: propidium iodide
SE: standard error
